# Insights into determinants of health-seeking behavior: a cross-sectional investigation in the Iraqi context

**DOI:** 10.3389/fpubh.2024.1367088

**Published:** 2024-06-26

**Authors:** Mohammed Mkhailef Hawi Al-tameemi, Kaveh Bahmanpour, Amjad Mohamadi-Bolbanabad, Yousef Moradi, Ghobad Moradi

**Affiliations:** ^1^Department of Health Care Management, School of Medical Sciences and Technologies Science and Research Branch, Islamic Azad University, Tehran, Iran; ^2^Department of Nursing, School of Medical Sciences, Islamic Azad University, Sanandaj, Iran; ^3^Social Determinants of Health Research Center, Research Institute for Health Development, Kurdistan University of Medical Sciences, Sanandaj, Iran

**Keywords:** health-seeking behavior, determinants, Iraq, HSB, health behavior

## Abstract

**Background:**

Health-seeking behavior (HSB) is a choice taken by an individual to maintain, achieve, or restore good health and prevent diseases. The purpose of this study is to examine the determinants of HSB among the Iraqi population.

**Methods:**

This cross-sectional study in the Rusafa and Karkh districts of Baghdad investigated determinants of HSB from 2022 to 2023. With a sample size of 993 participants meeting inclusion criteria, data were collected through a self-reported questionnaire, utilizing four indicators to measure HSB. The study employed various statistical methods especially logistic regression models, facilitated by Stata 17 software.

**Results:**

Results highlights that married individuals consistently have higher odds of having HSB compared to their single counterparts, with an odds ratio (OR) of 2.09 (95% confidence interval: 1.41–3.10). This relationship remains robust even after controlling for other variables. Furthermore, individuals with higher social class exhibit stronger connections to HSB, although the OR is 1.69 (95% CI: 0.47–6.13), indicating a wide confidence interval. Regarding underlying diseases and their duration, the results indicate that chronic diseases are associated with a higher likelihood of HSB, with an OR of 2.05 (95% CI: 1.35–3.11). Additionally, a longer duration of diseases in terms of years is also linked to a stronger association with HSB, with an OR of 2.86 (95% CI: 1.32–6.23).

**Conclusion:**

In conclusion, this work provides important insights into HSB. Married people are continuously more likely to engage in HSB than single people, highlighting the importance of customized interventions. Furthermore, persons from higher social classes have stronger ties to HSB, highlighting the importance of socioeconomic considerations. The link between HSB and chronic diseases, combined with longer disease durations, emphasizes the importance of early detection and thorough healthcare management. These findings give critical guidance for healthcare providers, marketers, and politicians developing effective initiatives to promote HSB.

## 1 Introduction

Health-seeking behavior (HSB) encompasses the actions individuals take to maintain, attain, or restore good health and prevent illness, ranging from seeking care at public or private health facilities to self-medication or refraining from utilizing available health services (1). This behavior is influenced by a multifaceted interplay of provider, patient, disease, and household factors (2), alongside various socioeconomic determinants including gender, age, social status, illness type, service accessibility, and perceived care quality (3, 4). Conflict situations, population displacement, and food scarcity further complicate HSB, posing challenges to both healthcare systems and individual well-being (5, 6). Iraq, enduring over four decades of conflict, war, and sanctions, has faced a deteriorating healthcare infrastructure and ongoing environmental hazards, contributing to adverse health outcomes (7).

The concept of intended HSB often involves seeking care through recognized healthcare channels, encompassing both public and private sectors, particularly prevalent in urban areas where the majority of health facilities are concentrated (1). Iraq’s healthcare landscape has gradually transitioned from predominantly hospital-centric, curative care to a primary focus on preventive care delivered through primary health clinics (PHCs) (8). Shabila et al. (9) identified four distinct HSB patterns among Iraqis, ranging from negative perceptions of private sector care to varying degrees of satisfaction and utilization of primary care services. While existing literature acknowledges these shifts, a comprehensive understanding of how evolving healthcare services influence HSB determinants remains elusive.

This study aims to address this gap by examining the factors shaping HSB in Iraq within the context of the country’s evolving healthcare delivery model, particularly emphasizing the shift toward PHCs and preventive care. By elucidating these dynamics, the research aims to provide insights crucial for healthcare providers, policymakers, and researchers, facilitating the development of targeted interventions to enhance equitable access to healthcare services in Iraq. Specifically, this study seeks to build upon existing literature by exploring novel aspects such as the impact of marital status and economic levels on HSB, thereby contributing new insights to the understanding of HSB in Iraq. By articulating these unique contributions, the study aims to firmly position itself within the existing literature while filling crucial gaps in knowledge regarding HSB determinants in the Iraqi context.

## 2 Methods

### 2.1 Study design

This cross-sectional study aimed to investigate the determinants of health-seeking behavior (HSB) in the districts of Rusafa and Karkh in Baghdad, Iraq, between 2022 and 2023. The study design adhered to the STROBE guidelines for reporting observational studies to ensure transparency and rigor in reporting (10). It was developed to provide a comprehensive understanding of HSB in these specific districts, considering their unique socio-economic, cultural, and infrastructural characteristics that may influence healthcare access and utilization.

### 2.2 Questionnaire development and pilot study

A structured questionnaire was developed based on the Pourreza model (11). The questionnaire underwent a rigorous adaptation process to suit the Iraqi context. To assess the clarity, appropriateness, and relevance of the questionnaire items, a pilot study was conducted with a small sample of participants from similar districts in Baghdad. The feedback from the pilot study participants was used to refine the questionnaire further, ensuring its suitability for the study population. The final version of the questionnaire was reviewed by 11 specialists in healthcare management and community medicine. Content validity ratio (CVR) and content validity index (CVI) were calculated for each question, with the CVR ranging between 63.6 and 81.8 for all questions and an average CVI of 0.83.

### 2.3 Participant recruitment and sampling

A multifaceted approach to recruiting participants was used to ensure a thorough understanding of HSB, including people who had recently used health services as well as those who had not. Initially, health centers providing primary health care services in the Rusafa and Karkh districts of Baghdad were selected. These centers served as the main sampling points for participant recruitment. Efforts were made to reach members of the community who may not have recently received health services. The results of the study were disseminated through a variety of channels, including community leaders and health care providers. Participants were selected from health centers that provided primary health care services to ensure that they were individuals who had recently received health care services. In addition, efforts were made to recruit individuals from the community who had not recently sought care to reflect a broad spectrum of HSB. This process was based on a multistage sampling design. The sample size was determined to be 664 individuals based on a 99% confidence level, a 50% response rate distribution, and an acceptable precision level of 0.05. However, during the actual data collection phase, a total of 993 individuals were enrolled to enhance the study’s statistical power and ensure a more robust representation of the population under examination.

### 2.4 Data collection

Trained data collectors, familiar with the local context and fluent in both Arabic and English, were responsible for administering the questionnaire. They underwent comprehensive training on the study objectives, ethical considerations, data collection procedures, and interview techniques. The training sessions included role-playing exercises and discussions to ensure consistency and standardization in data collection.

During the data collection phase, several measures were implemented to monitor and enhance data quality. Regular meetings were conducted with the data collectors to address questions, concerns, and ensure a shared understanding of the study objectives and procedures. Field supervisors were appointed to oversee the data collection process, providing ongoing support, guidance, and quality control. A random subset of completed questionnaires was selected for quality control purposes, and double data entry was performed to minimize data entry errors.

### 2.5 Ethical considerations

Ethical approval for the study was obtained from the Islamic Azad University Sanandaj (IR.IAU.SDJ.REC.1402.027). Informed consent was obtained from each participant before their inclusion in the study. Participants were provided with detailed information about the study objectives, procedures, potential risks, benefits, and their right to withdraw from the study at any time.

### 2.6 Statistical analysis

The collected data were entered into a statistical software program for analysis. Descriptive statistics were used to summarize the socio-demographic characteristics of the participants and the key variables related to HSB. Inferential statistics, such as chi-square tests, were performed to examine the associations between various determinants and HSB. Logistic regression analysis was specifically chosen to assess the relationship between the determinants and HSB, as it allows for the estimation of odds ratios (ORs) and the identification of significant predictors while controlling for confounding factors.

## 3 Results

In total, 993 individuals were involved in this research. The average age of the participants was 36.83 years with a standard deviation (SD) of 11.50 (36.83 ± 11.50). Among them, 56.6% (*n* = 562) were female, and 72% (*n* = 715) were married.

Regarding educational attainment, the distribution among participants was as follows: 278 individuals (28.0%) had completed middle school, 412 (41.5%) held a high school diploma, and 303 (30.5%) possessed a university degree. A majority of participants (58.3%) reported having a family size of four or fewer members (Table 1). In terms of socioeconomic status, 67.4% of participants fell into the middle-level category, while only 3.5% belonged to the high-level category (Table 1).

**Table 1:** Sociodemographic characteristics of participants (*N* = 993).

**Variable**	**Frequency**	**Percent**
**Gender**		
Male	431	43.4
Female	562	56.6
**Marital Status**		
Single/ Divorced/Widow/Widower	278	28.0
Married	715	72.0
**Educational**		
Unable to read and write to middle school	278	28.0
High school and diploma	412	41.5
University degree	303	30.5
**Family members**		
<=4	414	41.7
>4	579	58.3
**Socioeconomic level**		
Low	289	29.1
Middle	669	67.4
High	35	3.5

**Variable**	**Mean**	**SD** ^*^
Age (Years)	36.83	11.50
SD = Standard deviation		

All participants expressed a need for healthcare within the last 3 months. Among them, 867 individuals (87.1%) reported having sought HSB. Of these, 456 people (52.5%) sought treatment in the first days with mild symptoms of the disease, 369 individuals (42.5%) sought treatment at the onset of the disease and its symptoms, and 43 people (5%) sought treatment at a serious stage of the disease (Table 2).

**Table 2:** Health seeking behavior (HSB) of participants in the last 3 months (*N* = 993).

Variable	Frequency	Percent
**Health condition**		
Acute	585	58.9
Chronic	408	41.1
**Illness duration (years)**		
1	456	46.8
2	205	20.7
3	323	32.5
**Do you have attempted to treat your illness?**		
Yes	867	87.1
No	126	12.7
**How many days after the onset of disease symptoms did you get care from a provider? (*n* = 868)**		
The same day	207	23.9
The next day	250	28.8
Two days later	172	19.8
3–7 days	72	8.3
More than seven days	167	19.2
**At what stage of your disease have you gone to a provider? (*n* = 868)**		
In early stages and onset of symptoms mild	456	52.5
Incidence of disease and its symptoms	369	42.5
In serious stage of disease	43	5.0
**Where did you go to treatment in the first step? (*n* = 868)**		
General practitioner’s office, health center, emergency room	395	45.5
Clinic, private or governmental hospital	335	38.6
Pharmacy without a prescription but consultation with the pharmacist	62	7.1
Traditional healers, Pharmacy without a prescription and consultation with the pharmacist, I have not referred to	76	8.8
**Until what step of treatment have you completed your course of treatment according to provider (*n* = 868)**		
To received	454	52.3
To relieve the symptoms	334	38.5
Do not complete my course of treatment	80	9.2
**Have you ever had experience of care reception from health care centers? (Clinic, emergency room, doctor’s office, hospital, health centers)?**		
Yes	883	88.9
No	110	11.1

In response to the question “How many days after the onset of disease symptoms did you seek care from a provider?” the majority of participants sought care on the same day (23.9%) or the next day (28.8%). Additionally, 62 individuals (7.1%) initially visited a pharmacy without a prescription but consulted with the pharmacist as their first step for treatment. Only 52.3% of individuals who received treatment completed their course of treatment until recovery, while 47.9% did not complete their course of treatment (Table 2). The frequency with which people react to the question “Have you ever had an experience with care reception from a health care center?” (Clinic, emergency room, doctor’s office, hospital, or health center)?” Equal to 993. In this population, 883 people (88.9%) said yes (Table 2).

The chi-square test revealed a significant relationship between HSB and gender (*p* = 0.09), marital status (*p* < 0.001), educational qualification (*p* = 0.03), illness duration (*p* < 0.001), and health condition (*p* = 0.001), as indicated in Table 3. However, there was no significant relationship observed between HSB and family members or social class.

**Table 3:** Comparison of health-seeking behavior and demographic variables (*N* = 993).

Variable	HSB		Chi2*	
	Yes	No	*X*_2_	*p*-value
**Gender**				
Male	385 (89.33)	46 (10.67)	2.79	0.09
Female	482 (85.77)	80 (14.23)		
**Marital status**				
Single/Divorced/Widow/Widower	225 (80.94)	53 (19.06)	14.16	<0.001
Married	642 (89.79)	73 (10.21)		
**Educational qualification**				
Unable to read and write to middle school	252 (90.65)	26 (9.35)	6.88	0.032
High school and diploma	362 (87.86)	50 (12.14)		
University degree	253 (83.50)	50 (16.50)		
**Family members**				
<=4	365 (88.16)	49 (11.84)	0.46	0.49
>4	502 (86.70)	77 (13.30)		
**Social class**				
Low	261 (90.31)	28 (9.69)	4.26	0.11
Middle	574 (85.80)	95 (14.20)		
High	32 (91.43)	3 (8.57)		
**Health condition**				
Acute	493 (84.27)	92 (15.73)	11.85	0.001
Chronic	374 (91.67)	37 (8.33)		
**Illness duration (years)**				
1	386 (83.01)	79 (16.99)		
2	180 (87.80)	25 (12.20)	17.87	>0.001
+3	301 (93.19)	22 (6.81)		

*Chi-square test.

According to the results of univariate regression analysis, married individuals exhibit approximately twice the likelihood of having HSB compared to single individuals (OR: 2.07, 95% CI 1.40–3.04). After controlling for other variables, this association remained relatively stable (OR: 2.09, 95% CI 1.41–3.10). Additionally, individuals belonging to middle socioeconomic levels demonstrate a lower likelihood of HSB compared to those at low levels (OR: 0.64, 95% CI 0.41–1.01). Furthermore, individuals with a university level of education exhibit a decreased likelihood of HSB compared to those with a middle school education or less (OR: 0.52, 95% CI 0.31–0.86), a trend that persists even after adjusting for other variables (OR: 0.54, 95% CI 0.32–0.92) (Table 4). Regarding underlying diseases and their duration, the results indicate that chronic diseases are associated with a higher likelihood of HSB, with an OR of 2.05 (95% CI: 1.35–3.11). Additionally, a longer duration of diseases in terms of years is also linked to a stronger association with HSB, with an OR of 2.86 (95% CI: 1.32–6.23) (Table 4).

**Table 4:** Factors associated with HSB among Iraqi population (*N* = 993).

Variable	Health-seeking behavior Unadjusted *OR* (95% CI)	Health-seeking behavior adjusted *OR* (95% CI)
**Social class**		
Low	Reference	Reference
Middle	0.64 (0.41–1.01) *	0.74 (0.47–1.19)
High	1.14 (0.32–3.97)	1.69 (0.47–6.13)
**Gender**		
Female	Reference	Reference
Male	1.38 (0.94–2.04)	1.34 (0.90–2.00)
**Marital Status**		
Single/ Divorced/ Widow/Widower	Reference	Reference
Married	2.07 (1.40–3.04) ***	2.09 (1.41–3.10) ***
**Educational Qualification**		
Unable to read and write to Middle school	Reference	Reference
High school and Diploma	0.74 (0.45–1.23)	0.82 (0.49–1.38)
University degree	0.52 (0.31–0.86) *	0.54 (0.32–0.92) *
**Health condition**		
Acute	Reference	Reference
Chronic	2.05 (1.35–3.11) ***	0.97 (0.50–1.89)
**Illness duration (years)**		
1	Reference	Reference
2	1.47 (0.90–2.38)	1.44 (0.81–2.56)
+3	2.80 (1.70–4.59) ***	2.86 (1.32–6.23) **

* < 0.05 ** < 0.01 *** < 0.001. Adjusted variables: Gender, Marital Status, Educational Qualification, Social class, Health condition, Illness duration.

Adjusted variables: Gender, Marital status, Educational qualification, Social class, Health condition, Illness duration.

## 4 Discussion

This study engaged a cohort of 993 individuals from Iraq, focusing primarily on HSB among the Iraqi population. A significant finding was that 87.1% of respondents had engaged in HSB within the previous 3 months. Additionally, demographic variables such as marital status, social class, chronic disease and duration of the disease were identified as significant factors influencing HSB. Our research revealed statistically significant differences in HSB between gender. Khajeh et al. (12) reported that women exhibited higher levels of adherence to treatment regimens and expressed greater satisfaction with their healthcare experience. While the association between gender and treatment-seeking behavior was inconclusive, our findings suggest that men are more inclined to self-medicate, potentially influenced by gender-related factors. Existing literature on this topic presents conflicting results, with some studies suggesting higher rates of self-medication among women, while others report the opposite (12). In a different study, men reported self-medicating more frequently than women (13).

Notably, a comprehensive survey conducted in the United Kingdom revealed that women engage with their primary healthcare providers more than twice as frequently as men. Similar patterns were observed in mental health studies conducted in Canada and the United States. However, it is important to acknowledge that these gender discrepancies in healthcare utilization may not always reach statistical significance due to the heterogeneity of study methodologies and the diverse influences of socioeconomic factors on different gender groups (14). The evident gender disparities in healthcare utilization, as highlighted by extensive surveys in the UK and echoed in mental health research across North America, underscore the complex interplay of societal, cultural, and individual factors shaping HSB. These observations align with broader discussions on gender roles, where women may be socialized to prioritize health maintenance, while societal norms surrounding masculinity may discourage men from seeking medical care or openly discussing health concerns (15, 16).

The study underlines the importance of considering both sex and gender in healthcare utilization research. This approach allows for a better understanding of whether differences are explained by biological factors or indirect and consequently helps identify modifiable risk factors for unfavorable outcomes. The study also highlights the need for policy-makers to plan the measurement of sex at birth, gender identity, and gender-related variables in survey and patient registry developers to allow for more relevant, equitable, diversified, and inclusive future research (17–21).

The significant gender discrepancies in healthcare consumption, as revealed by a large survey in the UK and repeated in mental health research across North America, provide a nuanced view of how societal, cultural, and individual factors interact to determine HSB. The fact that women contact their primary healthcare providers more frequently than males shows that there may be discrepancies in health awareness, societal expectations, and goals for well-being (19–21). These findings are consistent with broader conversations about gender roles, in which women may be taught to prioritize health maintenance, while cultural norms around masculinity may contribute to men’s reluctance to seek medical care or openly address health concerns. Furthermore, socioeconomic factors exacerbate gender disparities in healthcare consumption. Economic gaps affect access to healthcare resources, insurance coverage, and the capacity to leave work for medical appointments. The complex interplay between gender and socioeconomic position highlights the importance of specialized interventions that address the unique obstacles faced by different gender groups. Furthermore, the variation in outcomes between studies emphasizes the need of examining different approaches and definitions of healthcare usage. To gain a thorough understanding of these differences, we must recognize not just the variation between gender groups, but also the complex interplay of cultural, economic, and individual factors impacting healthcare practices (22–28).

As societies grapple with the implications of these gender-related healthcare patterns, policymakers and healthcare providers are challenged to develop strategies that foster inclusivity and address the unique needs of diverse populations. Tailoring interventions to account for the intersectionality of gender with other social determinants, such as socioeconomic status, can pave the way for more equitable healthcare access and outcomes. Embracing a holistic and interdisciplinary approach will be essential in dismantling the barriers that contribute to gender disparities in healthcare utilization and working toward a more inclusive and responsive healthcare system.

Similarly, Patel et al. (29) discovered that registration in any government program, educational level, and poverty status were all significant predictors of HSB in the older adult residing in Shri Vasantrao Naik Government Medical College’s urban field practice area in Yavatmal, Maharashtra. According to Thompson et al. (30), there were gender disparities in how people sought medical assistance, with women indicating that they saw their primary care physician for physical and mental health concerns more frequently than males. This finding supports the notion that women are more concerned about their health than males. Patients were generally less likely to seek treatment for mental health difficulties than for physical ones. According to the regression analyses, age, disease prevention, physician trust, and chronic conditions were all important variables in explaining healthcare-seeking behaviors for mental health problems in both men and women (30).

Education level was identified as one of the variables affecting HBS. Education plays a crucial role in determining various aspects of healthcare behavior, including adherence to treatment programs, the propensity for self-medication, recognizing when to seek medical attention, and more. Research indicates that public education initiatives and the application of behavioral models can significantly reduce the prevalence of self-medication. Muriithi (31) found that educated individuals are more likely to opt for professional healthcare services over self-care practices. These findings are consistent with numerous other studies in the literature, indicating robust and consistent parameter estimations across various healthcare settings (31). Contrary to Sahn et al. (32, 33), it is noteworthy that the demand growth rate for public health facilities is larger than for private ones. Cisse (32) discovered that education positively affected healthcare demand. Hutchison et al. (34) discovered that educated women were more likely than illiterate women to seek medical treatment. The study suggests that educated individuals may tell the difference between actual and perceived health treatment quality by seeing the health practitioners’ credentials. As opposed to private clinics, where the qualification of the health workers is not readily recognized, a public health institution guarantees quality and trained health personnel (34). Muriithi (31)refutes the generally accepted belief that years of education diminish the likelihood of obtaining health care from a public health institution vs. self-treatment.

Furthermore, our data suggest that more than half (58.9%) of individuals had acute health issues. Health status was identified as one of the variables affecting HBS. The survey outcomes also demonstrated statistically significant differences in consumers’ perceptions of healthcare providers and overall HBS across categories of health problems. According to a 2019 study by Khajeh et al. (12), there was no association between the severity of the sickness and the frequency of clinic visits.

Interestingly, our research uncovered a counterintuitive trend regarding the negative link between disregarding therapy and the severity of the condition. Contrary to conventional expectations, individuals exhibited a reduced tendency to seek formal medical treatment when faced with more severe health issues. The authors of this study discovered numerous critical data that support their hypothesis about the impact of the developing healthcare delivery landscape on HSB among the Iraqi population. Specifically, a sizable proportion of participants reported receiving healthcare primarily through primary health clinics (PHCs) rather than traditional hospital-based systems. This shift to PHCs is consistent with Iraq’s healthcare system’s transition from curative to preventative treatment. Furthermore, the findings of this study suggested that people from higher social classes had stronger ties to HSB. This study implies that as healthcare delivery in Iraq has transitioned to preventative care via PHCs, people with more means and a higher socioeconomic standing may be better positioned to take advantage of these services. In contrast, persons from lower socioeconomic backgrounds may experience challenges to accessing PHCs and engaging in preventative treatment, thus contributing to HSB inequities. Furthermore, the found disparities in HSB by demographic parameters such as gender, marital status, and educational level highlight the influence of the changing healthcare landscape. These demographic discrepancies may reflect differences in access to and use of PHC services, emphasizing the importance of targeted interventions to achieve equitable access to preventative care across varied demographic groups. This finding prompts consideration of alternative explanations, such as the possibility that individuals seek therapy when an illness reaches a critical point but may opt for non-medical avenues, including traditional treatments. Another plausible explanation is that individuals may perceive their sickness as more severe than it actually is, leading them to bypass formal medical consultations in favor of alternative approaches.

Our findings have far-reaching implications for policy and practice, informing broader health policy in Iraq, particularly during post-conflict reconstruction. Understanding the factors of HSB found in this study might help drive the creation of tailored interventions to improve healthcare access and usage across the country.

First, policymakers might use our findings to select areas for intervention, with an emphasis on addressing the reported discrepancies in HSB across demographic groups. For example, campaigns could be designed to raise awareness and education regarding healthcare-seeking behaviors among people with lower educational attainment or socioeconomic level. Furthermore, focused efforts may be required to address cultural attitudes and norms that influence health-seeking decisions, particularly those involving traditional vs. formal medical treatments.

Furthermore, our findings emphasize the importance of integrated healthcare delivery systems that incorporate both traditional and modern healthcare methods. This acknowledgment can help to shape healthcare policies and initiatives that embrace culturally sensitive approaches and value traditional healers as vital partners in the healthcare system. In addition, in the context of post-conflict reconstruction, where healthcare infrastructure may have been badly affected, our findings highlight the critical need of restoring and enhancing primary healthcare services. Investments in primary care facilities and worker capacity building can improve access to basic healthcare services while also encouraging early detection and management of health issues.

Specific interventions could include community-based health education initiatives, targeted outreach efforts to vulnerable populations, and the formation of referral networks to ease access to specialized treatment when necessary. Furthermore, initiatives to strengthen healthcare finance systems, such as increasing health insurance coverage or establishing subsidies for important health services, can assist lower financial barriers to care access.

This study has several limitations. First, the cross-sectional design of our study limits our ability to determine causality between variables. Cross-sectional studies collect data at a single point in time, which precludes the assessment of temporal relationships between variables. As a result, while our study identified associations between HSB and various demographic and socioeconomic factors, we cannot infer causality. Future research should utilize longitudinal designs. Second, the self-reported nature of data collection may have introduced biases and limitations. Participants’ responses may have been influenced by social desirability bias, which occurs when people provide comments that they believe are socially acceptable rather than reflective of their actual behavior or experiences. In addition, recall bias may affect the accuracy of participants’ reports, especially those related to past health-seeking behaviors and experiences. In addition, relying on self-reported data may introduce measurement error because people may misinterpret survey questions or unintentionally provide false responses. To address these limitations, future research could use mixed-methods approaches that combine self-reported data with objective measures or observational data to provide a more complete picture of HSB. Validation studies could also be conducted to assess the accuracy of self-reported measures compared with objective measures of health care utilization.

## 5 Conclusion

In conclusion, this study offers insight on the intricacies of HSB among the Iraqi population, as well as the barriers within the current healthcare system that may prevent individuals from receiving adequate and quality care. The found HSB inequalities by gender, marital status, and educational level highlight the need of addressing socioeconomic inequities in healthcare consumption.

These findings have major implications for Iraqi healthcare policy and practice, especially given the country’s persistent healthcare issues. By identifying and addressing the factors that influence HSB, policymakers and practitioners can design more targeted and effective interventions to improve healthcare access and utilization. Such initiatives have the potential to improve health outcomes while also helping to develop Iraq’s healthcare system. Moving forward, efforts to address economic and educational inequities, as well as programs to raise awareness and education about healthcare-seeking behaviors, are critical for establishing a healthcare system that is accessible, egalitarian, and responsive to the needs of all Iraqi residents. We can work together to create a healthy future for Iraqis by implementing evidence-based solutions.

## Data availability statement

The original contributions presented in the study are included in the article/supplementary material, further inquiries can be directed to the corresponding author.

## Ethics statement

The studies involving humans were approved by this study was inspected based on ethical criteria and approved by the Islamic Azad University Sanandaj (with ethics code IR.IAU.SDJ.REC.1402.027). The studies were conducted in accordance with the local legislation and institutional requirements. Written informed consent for participation in this study was provided by the participants’ legal guardians/next of kin.

## Author contributions

MM: Writing – review & editing, Conceptualization, Data curation, Investigation, Methodology, Validation, Writing – original draft. KB: Writing – review & editing. AM-B: Writing – review & editing. YM: Writing – review & editing, Conceptualization, Data curation, Formal analysis, Investigation, Methodology, Resources, Supervision, Validation, Visualization, Writing – original draft. GM: Investigation, Methodology, Resources, Supervision, Validation, Visualization, Writing – review & editing.
